# Motor Coordination in Children: A Comparison between Children Engaged in Multisport Activities and Swimming

**DOI:** 10.3390/sports11080139

**Published:** 2023-07-25

**Authors:** Dušan Stanković, Maja Horvatin, Jadranka Vlašić, Damir Pekas, Nebojša Trajković

**Affiliations:** 1 Faculty of Sport and Physical Education, University of Niš, 18000 Niš, Serbia; nele_trajce@yahoo.com; 2 University of Zagreb Faculty of Kinesiology, 10000 Zagreb, Croatia; maja.horvatin@kif.unizg.hr (M.H.); jadranka.vlasic@kif.unizg.hr (J.V.); damir.pekas@kif.unizg.hr (D.P.)

**Keywords:** motor competence, physical activity, physical fitness, perceived competence

## Abstract

Motor coordination has a crucial role in various physical activities and sports, highlighting its significance in overall movement proficiency and performance. This study aimed to compare motor coordination in children engaged in multisport versus swimming activities. The participants of this study included 180 boys and girls (girls = 87) aged 8.25 years ± 0.89. A total of three groups were included: group 1 consisted of inactive children, group 2 included children participating in swimming, and group 3 included children enrolled in multisport. Motor coordination was assessed using the Kiphard–Schilling body coordination test, evaluated by motor quotient (MQ): walking backwards, hopping for height, jumping sideways, and moving sideways. Additionally, a total motor quotient (Total MQ) was calculated based on the performance in all four tests. ANOVA revealed a significant difference in Total MQ and all subtests between the groups (*p* < 0.01). A significant difference in Total MQ was found not only between the inactive and multisport groups (Diff = 19.8000; 95%CI = 13.1848 to 26.4152; *p* = 0.001) but also between the multisport and swimming groups (Diff = 12.8000; 95%CI = 6.3456 to 19.2544; *p* = 0.001). In conclusion, the results revealed that children involved in multisport activities exhibited significantly better motor coordination compared to both the swimming group and the inactive group. Therefore, to enhance the growth of motor coordination abilities, it is crucial that parents, instructors, and coaches encourage kids to engage in multisport physical activities on a daily basis.

## 1. Introduction

Numerous experts in the field of motor development [1,2,3] state that knowing basic motor skills is very important if a person wants to remain active throughout their life. Regarding the importance of forming stable basic motor skills, Clark and Metcalf [1] note that the inability to perform basic locomotor and manipulative skills will result in limited opportunities for physical activity as children grow due to inadequately developed prerequisites. Motor coordination is considered to be one of the most important abilities in early childhood. This was confirmed by Lopes et al. [4] who stated the development of motor coordination should be a key strategy in childhood for long-term obesity prevention and physical activity promotion. Also, controlling two or more body parts simultaneously or moving the entire body in space at once requires motor coordination [5]. Motor coordination is fundamental both in the acquisition and development of specialized movements and techniques in daily life and sport. Additionally, Lubans et al. [6] have stated that motor competence is positively associated with health and increased physical activity.

Although researchers have used the Körperkoordinationstest für Kinder (KTK) [7] battery in their research as an instrument to assess fundamental motor skills, gross motor skills, dynamic balance, and stability [8], the KTK is the most commonly used test battery for assessing the coordination of the whole bodies of children aged 5–14 years [9]. The fact that the KTK is considered a suitable battery for different purposes in different fields such as educational composition, sports, health fields, medicine, and biomechanics can be interpreted as a strength of the KTK [7]. A great majority of motor coordination studies have been focused on its correlation with body mass index, plyometric training, physical activity, and fitness [4,10,11,12,13]. Iivonen et al. [7] consider the fact that only nine studies have determined the validity or reliability of the KTK in target populations to be undesirable. Also, few studies have examined the effects of intervention or treatment on motor outcomes measured by the KTK [14,15,16].

It is well known that children develop motor coordination by the maturation process [2]. However, the best possible form of motor coordination could be achieved with the appropriate training. This was confirmed by Fransen et al. [17] who stated that spending many hours in various sports is important for the proper development of gross motor coordination. Additionally, Jaakkola et al. [18] also stated that sport-specific training could enhance motor coordination in children, but it depends on the sport in which they are enrolled. Regarding the engagement of children in different sport activities (multisport), Popović et al. [19] found that children involved in multisport activities have better results in motor coordination than children who were playing only football. However, there is still a lack of research comparing the impact of different types of sports on motor coordination in children. Additionally, studies that investigated the impact of water sports on motor coordination are lacking. According to author’s knowledge, only one study [20] has compared the influence of swimming and soccer on gross motor development. The results in this study showed a positive impact of both swimming and soccer in motor proficiency. However, despite the KTK test battery, the test of gross motor development 2 (TGMD-2) was used. We decided to include swimming since this sport is one of the most popular among children, and children often specialize in the earliest phases of their childhood. Moreover, motor coordination is one of the essential skills for swimming because good motor coordination enables swimmers to move their limbs and body in a synchronized manner, allowing them to swim efficiently and effectively.

The findings of the study indicate a preferable program for developing motor coordination in children. Therefore, the purpose of this study was to determine the differences in motor coordination between inactive children, children enrolled in multisport activities, and children enrolled in swimming. It was hypothesized that the group of children engaged in multisport activities would show better gross motor coordination than the other two groups. 

## 2. Materials and Methods

### 2.1. Participants

The sample consisted of 180 participants including both sexes (boys = 93; girls = 87) aged 8.25 years (SD ± 0.89) and was divided into three groups according to their involvement in the physical activity program (inactive, multisport, and swimming). Inactive children included participants who were not engaged in any form of additional organized physical activity except compulsory PE and are from the territory of southern Serbia (boys = 28, girls = 30; mean age = 8.89 ± 0.69 years). The second group included children who had been engaged for at least one year in swimming sessions (boys = 33, girls = 26; mean age = 8.39 ± 0.91 years). The third group included children who had been engaged for at least one year in multisport activities (boys = 32, girls = 31; mean age = 8.18 ± 0.68 years). Additional inclusion criteria were that children had to have a minimum of 2 days per week of PE lessons and could not to be involved in other organized sport activity. The participants were included in regular primary school education in the territory of Serbia, where they had the opportunity to participate voluntarily in the measurement. Ethical approval for the study was obtained from the institutional ethics committee from the University of Novi Sad (ref no: 13/1042). Their parents or guardians signed inform consent for participation. 

### 2.2. Procedures 

The multisport activities consist of different sports (football, basketball, volleyball, etc.) and activities (strength, obstacle course, etc.) with no swimming sessions. This concept provides training and development programs to help children improve their skills in different sports. Additionally, the program is designed to ensure that the skills being taught are aimed at improving a child’s motor skills, strength, and coordination, as well as other skills like teamwork, concentration, and listening. Multisport training consists of different sports and indoor activities in a fully equipped gym two days per week for ~60 min with no swimming sessions. Sessions consisted of multiple sports activities, and exercises were led by educated PE teachers. Every week, activities were focused on a skill or group of skills from one of the three gross motor skill categories: stability (trunk strength), locomotion (running, hopping, and skipping), or manipulation (ball skills). Additionally, children were introduced every week to the most important elements of team and individual sports. In the first session in a week, children were introduced to motor skills, and movement concepts were added in the second session at the end of the week. Later in the program, skill patterns were incorporated into activities. Swimming sessions were performed two days per week at the city swimming pool for ~60 min as well. Swimming group sessions were based on learning and stroke development (freestyle, breaststroke, etc.). The inactive group of children was included only in regular PE lessons, two days per week for ~45 min. Both multisport and swimming groups had regular PE lessons. 

Standardized protocols were used to assess body height and body mass using a portable stadiometer [21] and a digital scale [22]. The measurements were taken down to the nearest 0.1 cm and 0.1 kg.

The testing was conducted by two PhD students and two professors [23]. Tests were conducted in the school gym, in the morning hours, and on a single day. The schedule of the tests was as follows: First, anthropometric measurements of body height and body weight were taken in two groups. Then, the children were divided into four groups and distributed in such a way that each group was on one KTK subtest: walking backwards on beams, lateral jumps, one-legged jumping over obstacles, and moving platforms. After each group’s test was completed, they rotated clockwise according to the written schedule. When all groups completed all four tests, the measurements were completed. The tests were placed in the corners of the room so that there was a logical direction of the rotation of the groups.

### 2.3. Measures

Motor coordination was evaluated with the Kiphard–Schilling body coordination test [24] and the Körperkoordinationstest für Kinder (KTK). The KTK consists of four subtests: (1) walking backwards (WB) on a decreased-width balance beam; (2) moving sideways (MS) using wooden boards; (3) hopping for height (HH) by jumping off a single leg over a foam obstacle; (4) jumping sideways (JS) with both legs moving side-to-side. According to the raw scores of these four subtests, an age- and sex-specific percentile rank was calculated using normative data of 1228 normally developing German children [24]. Raw scores for each test are transformed using sex- and age-specific tables derived from the original German sample to improve comparability independent of age and sex or converted to an overall motor quotient. The raw score for each test was retained for analysis in the current study.

The “motor quotient” (MQ), a global indicator of MC adjusted for age and gender, was calculated using the four items and used as an indicator of MC. The MQ allows an assessment of the gross motor development in the following categories: ‘severe motor disorder’ (MQ 56–70, percentile 0–2), ‘moderate motor disorder’ (MQ 71–85, percentile 3–16), ‘normal’ (MQ 86–115, percentile 17–84), ‘good’ (MQ 116–130, percentile 85–98), and ‘high’ (MQ 131–145, percentile 99–100).

#### Statistical Analysis

Data are expressed as means ± standard deviations. Before using parametric tests, the assumption of normality was verified using the Kolmogorov–Smirnov test. Differences between the groups of children who were inactive, the children who had been engaged in multisport activities, and the children enrolled in swimming programs were determined using one-way ANOVA. A post hoc analysis for multiple comparisons was used to compare the test scores of the three subgroups (single-sport participants involved in swimming; multisport participants involved in multisport; and inactive children). The level of significance was set at *p* < 0.05. All statistical analyses were performed in SPSS statistical software (SPSS 23.0, IBM Inc., Chicago, IL, USA).

## 3. Results

The mean and SD are shown in Table 1 for each subtest and the total MQ. Moreover, Table 1 shows the results of ANOVA for the difference in the KTK test battery between inactive children, children enrolled in swimming, and children enrolled in multisport activities. 

**Figure 1:**
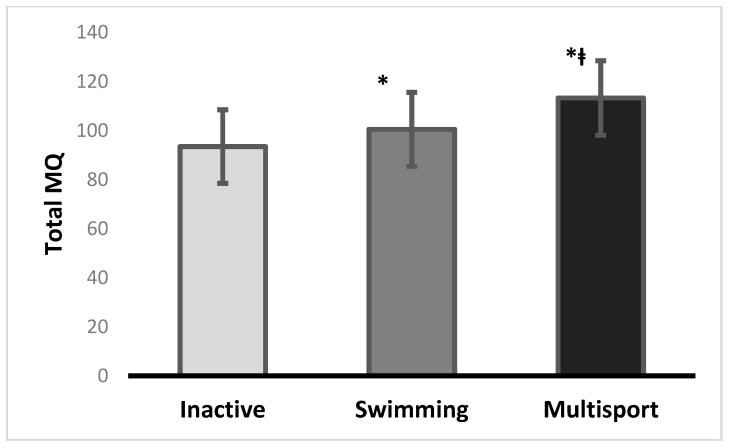
Bar chart of differences between groups in Total MQ. * Different from inactive; ⱡ different from swimming.

In the walking backwards test, the inactive group and the swimming group showed a significant difference in mean scores, with the swimming group having a higher mean score (Diff = 11.3100; 95%CI = 6.0447 to 16.5753; *p* = 0.001). The inactive group and the multisport group also showed a significant difference in mean scores, with the multisport group having a higher mean score (Diff = 8.8100; 95%CI = 3.4380 to 14.1820; *p* = 0.001. For the hopping for height test, the swimming group and the multisport group showed a significant difference in mean scores, with the multisport group having a higher mean score (Diff = 5.8000; 95%CI = 0.5586 to 11.0414; *p* = 0.0261). In the jumping sideways test, significant differences between the groups were the same as in the hopping for height test. In the moving sideways test, there was no significant difference in mean scores between the multisport group and swimming group. The inactive and multisport groups also showed a significant difference in mean scores, with the multisport group having a higher mean score (Diff = 8.1000; 95%CI = 5.7205 to 10.4795; *p* = 0.001). For Total MQ, there was a significant difference in mean scores between the inactive and swimming groups (Diff = 7.0000; 95%CI = 0.5162 to 13.4838; *p* = 0.0309). The swimming group had a higher mean score, indicating better motor coordination performance compared to the inactive group. A significant difference in Total MQ score was found between the inactive and multisport groups (Diff = 19.8000; 95%CI = 13.1848 to 26.4152; *p* = 0.001). Finally, the multisport group had a significantly higher Total MQ score, indicating the highest motor coordination performance among the three groups (Figure 1). 

## 4. Discussion

The present study investigated the comparison of motor coordination in children engaged in multisport activities, children engaged in swimming, and those who were inactive. In agreement with this study’s hypotheses, the main findings of the current study were that children who were engaged in multisport activities had better total motor coordination scores than children who were participating in swimming classes and those who were inactive.

Individuals with good motor coordination may have improved sports performance, better fine motor skills, and overall enhanced physical function, highlighting the importance of motor coordination in daily life [25]. The current findings suggest that multisport activities provide the highest results in motor coordination in children conducting the KTK. One possible explanation for this is that multisport activities expose children to a wider range of movement patterns and physical challenges, leading to a more diverse set of motor skills. In contrast, swimming may provide a specialized set of improvements that are focused on muscular endurance and muscular strength but not on coordination [26]. This may limit the development of overall motor coordination skills in children who only engage in swimming activities.

Based on our study, when observing the motor quotient for the moving sideways test alone and not the Total MQ, we found similar outcomes between the swimming and multisport groups. This finding is consistent with the results of the study by Fransen et al. [17], which showed no differences in physical fitness and gross motor coordination between children who participated in swimming and those who participated in gymnastics. This knowledge can suggest that participating in either swimming or multisport activities may provide similar benefits for motor coordination in children when considering only the moving sideways test in which they walked on wooden boards. This is an important finding as it suggests that children may be able to achieve similar levels of motor coordination by participating in different types of physical activities in particular coordination tests. In our study, only one test (walking backwards on a beam) showed higher scores in the swimming group than in the multisport group, which are trivial and not significant. Moreover, the multisport group has demonstrated to be the most advanced regarding Total MQ, which is a reliable and valid measure for comprehensive motor coordination. Some studies have found that, while swimming can improve cardiovascular fitness [27,28], it may not be as effective as other sports at improving specific aspects of motor coordination. Moreover, the study by Fransen et al. [17] found that children who participated in gymnastics had better Total MQ than those who participated in swimming. The authors suggested that this may be due to the specific demands of gymnastics training, which requires a high level of balance, coordination, and spatial awareness. Similarly, Jaakkola et al. [18] showed that young gymnasts had better results than hockey players and swimmers in the walking backwards tests and had an overall better result than hockey players in all coordination tests. De Lima and Castilha [29] presented interesting results showing that a group of swimmers had worse results than a group of non-swimmers (KTK test) who had regular physical education lessons. The authors explained these results by the fact that swimming lessons did not train this type of coordination. Compared to the aforementioned study, inactive children in the current study had the lowest scores in all subtests, as well as in total motor coordination scores. Therefore, although studies have shown swimming groups to have the lowest level of coordination, children engaged in swimming still show higher levels of motor coordination than inactive children. This highlights the importance of physical activity for the development of motor coordination skills in children.

It is well documented that frequent physical activity has a positive impact on motor coordination development and overall physical health in children [30]. However, this also highlights the importance of developing motor coordination in early childhood due to its significant correlation with health. Encouraging an improvement in the level of coordination can help make children healthier and more active. This was confirmed in a longitudinal study by Lima et al. [31]. Moreover, it is also essential that children under the age of 12 are encouraged by their parents and training experts to participate in multiple sports [17] other than just their ‘‘main sport’’ to improve motor coordination and career development in general. 

In the current study, the differences in motor coordination identified between the children engaged in multisport activities, children engaged in swimming, and inactive children could be aligned with a logical association with the intensive and specific training of the different sports in the multisport activities. Specifically, gymnastics is included in the multisport activities, which could significantly contribute to the differences. Moreover, the abovementioned differences were mainly due to the fact that the structured multisport program was based on motor coordination, stability, and locomotor and object control skills and activities. The importance of multisport activities for children has been confirmed previously [19,32].

It is important to note that the current study has several limitations. First, the study was cross-sectional in nature, and therefore, it is not possible to determine the relationship between physical activity and motor coordination development. Future longitudinal studies are needed to investigate the long-term effects of multisport activities and swimming on motor coordination development in children. Children who were included in the study were self-selected or recruited by their parents or coaches. This means that they may have had a particular interest in sports or physical activities, which could have influenced their motor coordination performance. The study did not control this bias. The study did not collect information on the attendance frequency and intensity of the physical activity in which the children were engaged. It is possible that children who were engaged in multisport activities performed sessions at a higher intensity than those who only swam or were not included in additional activities. This information could provide a better understanding of the impact of multiple sports or swimming on motor coordination development. The study used a single measure, the KTK test, to assess motor coordination. While the KTK test is a validated measure, it only assesses a limited number of motor coordination skills. Future studies could include additional measures to assess a wider range of motor coordination skills. The study sample consisted of children from a single geographic location and socioeconomic background. This limits the generalizability of the findings to other populations. Future studies could include a more diverse sample of children to improve the generalizability of the findings. However, the study included both male and female children, which increases the generalizability of the findings to both sexes, and the study included a relatively large sample size of 180 children, which increases the statistical power of the findings.

## 5. Conclusions

In conclusion, the children enrolled in multisport activities tended to show the highest levels of motor coordination compared to children who were engaged in swimming or who were inactive. Moreover, the swimming group had higher levels of motor coordination than the inactive group. The results emphasize the importance of multisport activities for the development of motor coordination skills in children. Therefore, it is of great importance that younger children (before the age of 10) are encouraged by their coaches, parents, and teachers to participate in different sports as opposed to just one sport or being inactive. 

## Figures and Tables

**Table 1:** Results of motor coordination tests in inactive children, children engaged in swimming, and children engaged in multisport activities.

	Inactive	Swimming	Multisport	*p*
Walking backwards	33.0 ± 13.9	44.0 ± 10.9 ^a^	42.9 ± 11.5 ^a^	0.001
Hopping for height	38.4 ± 13.8	40.7 ± 10.6	46.5 ± 11.6 ^ab^	0.001
Jumping sideways	51.5 ± 11.6	53.7 ± 11.1	63.8 ± 14.5 ^ab^	0.001
Moving sideways	34.5 ± 4.7	40.4 ± 5.8 ^a^	42.6 ± 7.5 ^a^	0.001

*p* < 0.05; ^a^ different from inactive group; ^b^ different from swimming group.

## Data Availability

The presented data can be provided by the authors upon request.
